# Diel-scale temporal dynamics in the abundance and composition of pollinators in the Arctic summer

**DOI:** 10.1038/s41598-020-78165-w

**Published:** 2020-12-03

**Authors:** Leana Zoller, Joanne M. Bennett, Tiffany M. Knight

**Affiliations:** 1 Institute of Biology, Martin Luther University Halle-Wittenberg, Am Kirchtor 1, 06108 Halle (Saale), Germany; 2 German Centre for Integrative Biodiversity Research (iDiv) Halle–Jena–Leipzig, Puschstrasse 4, 04103 Leipzig, Germany; 3 Centre for Applied Water Science, Institute for Applied Ecology, Faculty of Science and Technology, University of Canberra, Bruce, ACT 2617 Australia; 4 Department of Community Ecology, Helmholtz Centre for Environmental Research-UFZ, Theodor-Lieser-Straße 4, 06120 Halle (Saale), Germany

**Keywords:** Community ecology, Animal behaviour, Ecology

## Abstract

Our understanding of how pollinator activity varies over short temporal scales is limited because most research on pollination is based on data collected during the day that is then aggregated at a larger temporal scale. To understand how environmental factors affect plant–pollinator interactions, it is critical that studies include the entire diel cycle to examine patterns and processes that cause temporal variations. Further, there is little information from the Arctic, where environmental conditions that influence pollinator activity (e.g. temperature and solar radiation), are less variable across the diel cycle during the summer compared to locations from lower latitudes. We quantified abundance, composition and foraging activity of a pollinator community in Finnish Lapland at a diel scale over two summers, one of which was an extreme heat year. Pollinators showed a robust pattern in daily foraging activity, with peak activity during the day, less to no activity at night, and an absence of typically night active Lepidoptera. Abundance and composition of pollinators differed significantly between the years, possibly in response to the extreme heat in one of the years, which may particularly harm muscid flies. Our results showing strong diel and interannual abundance patterns for several taxa of pollinators in the Arctic summer have important implications for our understanding of temporal dynamics of plant–pollinator interactions.

## Introduction

Approximately 90% of angiosperms depend on animal pollination to some extent, making pollination a vital ecosystem service for the maintenance of plants^1^. The abundance and composition of pollinators, and thus the services they provide, are known to change across space and time. In order to understand and predict how environmental factors influence plant–pollinator interactions, it is critical that studies examine the patterns and processes that cause spatial and temporal variation^2^. Studies documenting pollinator communities are often based on data aggregated at large temporal scales, typically across weeks or entire seasons^3^. However, pollinator abundance and composition can vary considerably, even over short periods of time, such as a 24-h period^4^. Understanding fluctuations in pollinator abundance and composition on a 24-h temporal grain is of great importance because pollinator behaviour on a diel scale can affect the pollination success of plants. For example, plant reproductive output is higher when pollinator visitation and plant diel patterns (e.g. timing of anthesis, stigma receptivity or production of floral resources) are synchronised^5^. Hence, activity of pollinators during the daily cycle can provide a mechanistic understanding of the processes that take place at broader temporal scales. Currently, our understanding of diel patterns in the abundance and composition of pollinators is limited by a lack of nocturnal observations^4^, and a lack of information from high latitude locations in which summers experience constant daylight.

The diel foraging activity patterns of anthophilous insects are affected by factors which vary throughout the day. These factors include biotic ones, such as availability of plant resources^6^, predation and competition^5, 7^, as well as abiotic factors. Temperature, solar radiation and wind speed are the most important abiotic factors determining insect activity^5, 8, 9^. Butterflies for example derive their body heat almost exclusively through absorbing direct sunlight, hence their activity largely depends on solar radiation^8, 10^. Wind speed influences insect activity, since high wind speeds increase convective cooling and can cause navigation problems, especially for small animals^11^.

Abiotic factors that determine insect activity vary with latitude. In the Arctic Summer, a typical 24-h period has lower variation in both solar radiation and temperature compared to lower latitudes. There have not been any Arctic studies examining how the abundance and composition of pollinator communities change across the 24-h time period. However, it is known that some groups still have diel activity cycles. For example, bumblebees do not utilize the entire 24-h period for foraging during the Arctic summer, even though abiotic conditions, such as temperature and brightness, should allow them to do so. Instead they express a robust diurnal rhythm^12^ due to their intrinsic biological clocks^5, 13^. Flower visiting flies observed in south-western Norway show peak flower visitation activity during noon and no activity during the night^9^. Similarly, moths retain a distinct diel periodicity during the Arctic summer and are active at night, despite the ambient light being at levels that would inhibit activity in their relatives from lower latitudes^14^. This leads us to the expectation that we will find changes in foraging activity across the 24 h, even in the Arctic Summer.

Temperature is the most important determinant of activity of flying insects in the Arctic^15^, but to date, little is known about thermal tolerances of specific genera or species of Arctic pollinators. The primary orders of pollinators at high latitudes are Diptera, Hymenoptera and Lepidoptera, and the primary families are muscid flies (family Muscidae), syrphid flies (family Syrphidae) and Apidae (mainly represented through the genus Bombus). In recent review of thermal tolerances of 2133 organisms, none on these families were represented^16^. However, it has been proposed that flies in Arctic Alaska have a temperature optimum of around 13 °C and might be particularly sensitive to increased temperatures^17^. We expect that orders and families of pollinators might differ in their diel activity patterns, possibly due to differences in temperature sensitivity. But due to limited thermal tolerance information, we cannot make any more specific hypotheses.

During the peak flowering period in two Arctic summers we assessed the abundance and community composition and foraging activity of pollinators across a 24-h cycle. Specifically, we sampled the pollinator community in Lapland, 120 km north of the Arctic Circle. We predicted that the abundance, composition and activity of pollinators would change across the 24-h cycle, and that the abundance of pollinators would be highest in the middle of the day when temperatures are also high. We were fortunate to sample two very different years, one with temperature close to baseline conditions for the region and another that represents a mean temperature anomaly of over + 5 °C^18^.

## Results

### Abiotic factors

July 2018 was the hottest July in Finland since the records began in the early twentieth century^19^. Lapland experienced an unprecedented mean temperature anomaly of + 5 °C from the 1981 to 2010 July average of 14.1 °C^18^. Comparatively, temperatures in 2019 were close to average (Table 1). The mean temperature during the 2018 sampling rounds was significantly higher than in 2019. Means of other abiotic factors considered to influence pollinator activity (i.e., wind speed, global solar radiation) did not significantly differ between the two sampling years (Table 1, Supplementary Fig. S1). The mean density of flowering units (number of flowers or inflorescences observed per 30 × 2 transect) did not differ between the years (in 2018 = 3204, in 2019 = 2491; t = 0.391, p = 0.698), but there were differences in the identities of the seven most visited plant species across the years (Supplementary Fig. S2, Supplementary Table S1).

**Table 1:** Abiotic factors for the month of July in our two sampling years.

Abiotic factors	July 2018	July 2019	t	df	p
Monthly mean temperature (°C)	19.5	13.4			
Temperature anomaly (°C)	+ 5.4	− 0.7			
Max. sampling temperature	31.5	20.5			
Min. sampling temperature	11.7	5.6			
Mean temperature (°C)	22.7	12.5	8.400	72.225	**< 0.001**
Mean global solar radiation (W/m^2^)	1.319	197.78	1.246	77.531	0.216
Mean wind speed (m/s)	1.57	1.60	0.100	73.479	0.921

The data are provided by the Finnish meteorological Institute (FMI) and were recorded at the nearest available weather station to our site. Degrees of freedom (df), t- and p-values from t-tests comparing the mean values of air temperature, global solar radiation and wind speed recorded during our samplings across years are presented. Significant effects are printed in bold.

### Abundance and composition of pollinators

**Figure 1:**
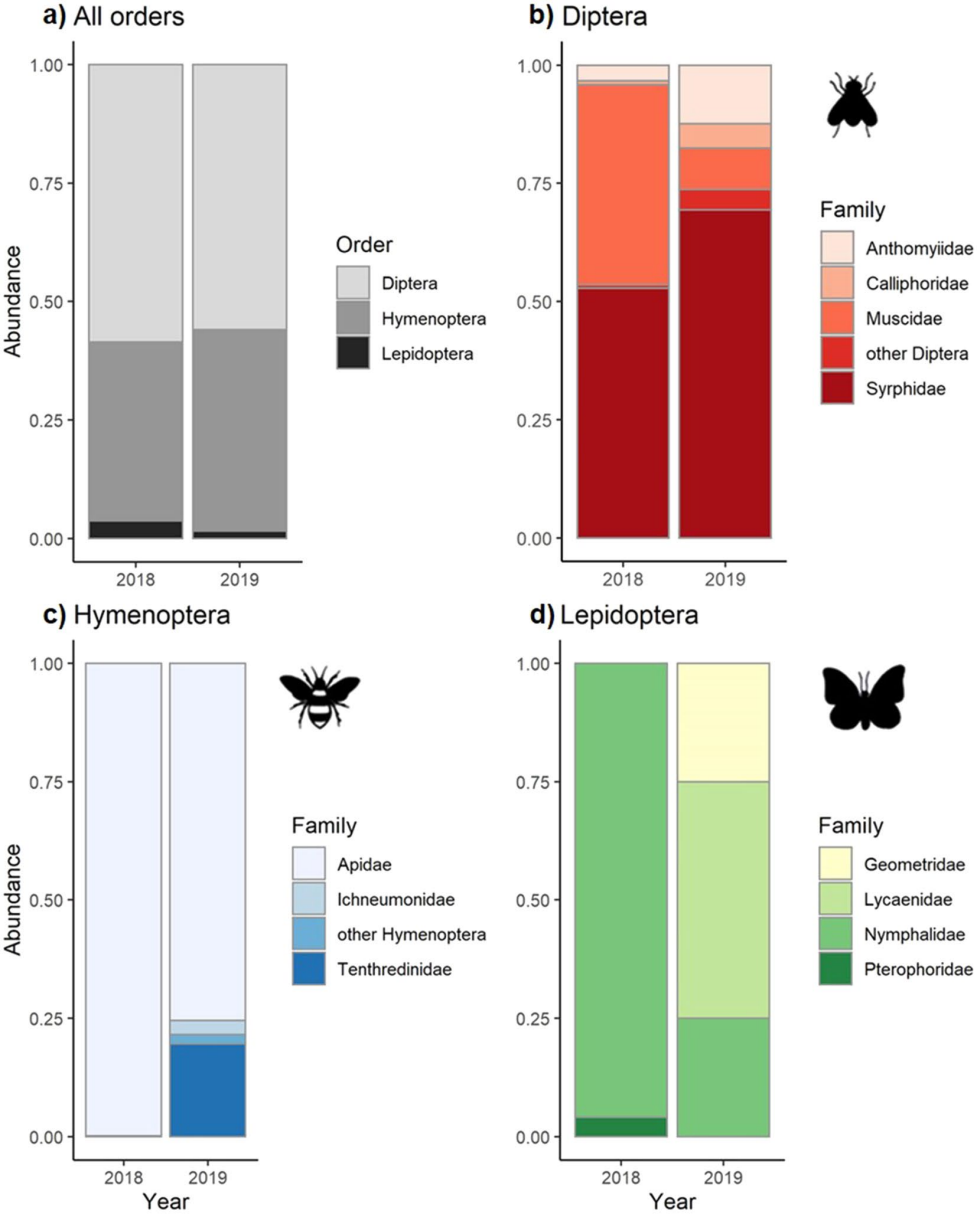
Relative abundance of pollinators for the two sampling years 2018 and 2019. (**a**) Relative abundance of the orders Diptera, Hymenoptera and Lepidoptera. (**b**) Relative abundance of each family among the Diptera. (**c**) Relative abundance of each family among the Hymenoptera and (**d**) relative abundance of each family among the Lepidoptera.

Across both sampling years we observed 1581 flower visitors from 19 families on 20 plant species. Ten families were observed in 2018, two of them exclusively. Sixteen families were recorded in 2019, nine of them exclusively. In both sampling years, Diptera was the most abundant order (58.57% of observations in 2018 and 55.89% in 2019), while Lepidoptera was the least abundant (3.72% and 1.52% respectively, Table 2). On the family level, Syrphidae represented 30.96% of total observations (52.85% of Diptera) in 2018 and 36.12% of all observations (64.63% of Diptera) in 2019, making them the most abundant Diptera family in both years. Muscidae made up 24.81% of all observations (42.36% of Diptera) in 2018, making them the second most abundant Diptera family in that year. In 2019, Muscidae represented 4.56% of all observations (8.16% of Diptera). Anthomyiidae made up 6.4% of all observations in 2019 (11.56% of Diptera), making them the second most abundant Diptera family in that year, while in 2018, they made up 1.97% of all observations (3.37% of Diptera). Apidae was the most abundant hymenoptera family in both 2018 and 2019, representing 37.63% of all observations in 2018 (99.8% of Hymenoptera) and 29.29% in 2019 (68.75% of Hymenoptera, Table 2, Fig. 1). Overall abundance of pollinators was 66.7% higher in 2018 (the record hot year) compared to 2019, and the abundance of most families differed significantly between the two sampling years (Table 2). Muscidae experienced the strongest change in relative abundance, with 20.25% lower abundance in 2019 compared to 2018 (Fig. 1, Table 2). The community composition was significantly different between the two sampling years (PERMANOVA: F = 14.869, R^2^ = 0.667, p = 0.01, Supplementary Fig. S3).

**Table 2:** Absolute and relative abundances, as well as the interannual relative difference in total abundance, for three orders of pollinators and the most abundant families within each order.

Taxon	Absolute abundance		t	df	p	Relative abundance of families within order		Relative abundance of taxa within year		Relative difference in abundance between years
	2018	2019				2018	2019	2018	2019	2018–2019
All taxa	**1318**	**263**	4.47	44.75	**< 0.001**					− 66.72
**Diptera**	**772**	**147**	3.97	27.15	**< 0.001**			**58.57**	**55.89**	**− 2.68**
Syrphidae	408	95	5.23	7.29	**0.001**	52.85	64.63	30.96	36.12	+ 5.16
Muscidae	327	12	9.40	6.43	**< 0.001**	42.36	8.16	24.81	4.56	− 20.25
Anthomyiidae	26	17	0.68	5.19	0.524	3.37	11.56	1.97	6.46	+ 4.49
**Hymenoptera**	**497**	**112**	3.07	6.09	**0.021**			**37.71**	**42.59**	**+ 4.88**
Apidae	496	77	7.13	6.44	**< 0.001**	99.8	68.75	37.63	29.28	− 8.35
Tenthredinidae	0	20	–	–		0	17.86	0	7.60	+ 7.60
**Lepidoptera**	**49**	**4**	2.27	7.31	0.056			**3.72**	**1.52**	**− 2.20**
Nymphalidae	47	1	–	–	–	95.92	25	3.57	0.38	− 3.19

Absolute abundance refers to the total number of observed individuals in each taxon. Relative abundance of families within orders describes the percentage of a family within the order. Relative abundance of taxa within a year represents the percentage of each taxon in relation to all observed individuals in a year. Only families of which at least 10 individuals were recorded are presented. Degrees of freedom (df), t- and p-values from the t-tests comparing the mean abundance of each taxon in 2018 and 2019 are presented. Significant effects are printed in bold.

### Diel foraging activity

**Figure 2:**
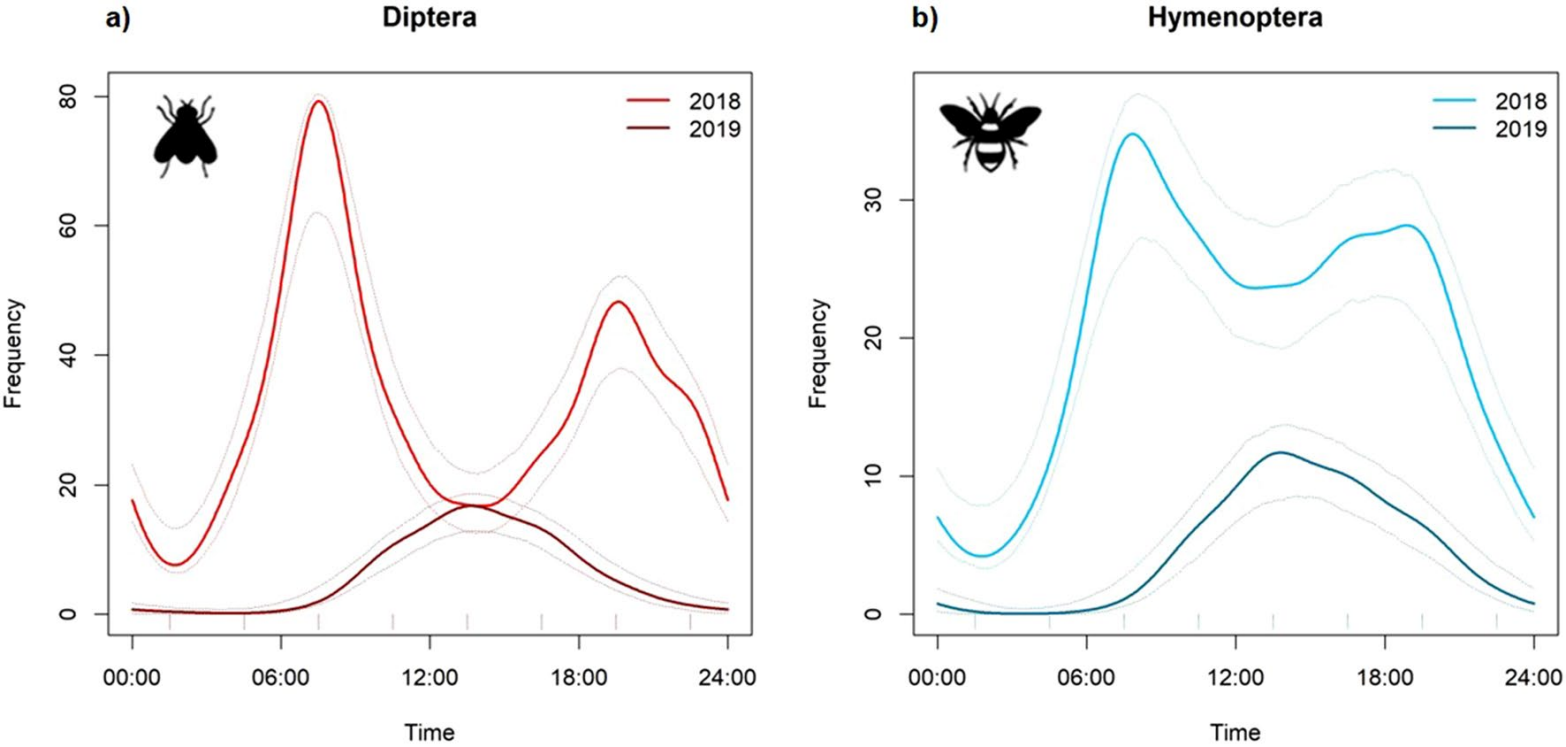
Activity patterns of the two most abundant orders of pollinators. Curves represent fitted circular kernel distributions of (**a**) Diptera and (**b**) Hymenoptera across the diel cycle and for the two sampling years 2018 and 2019. Dashed lines represent bootstrapped 95% confidence intervals of the activity models.

**Figure 3:**
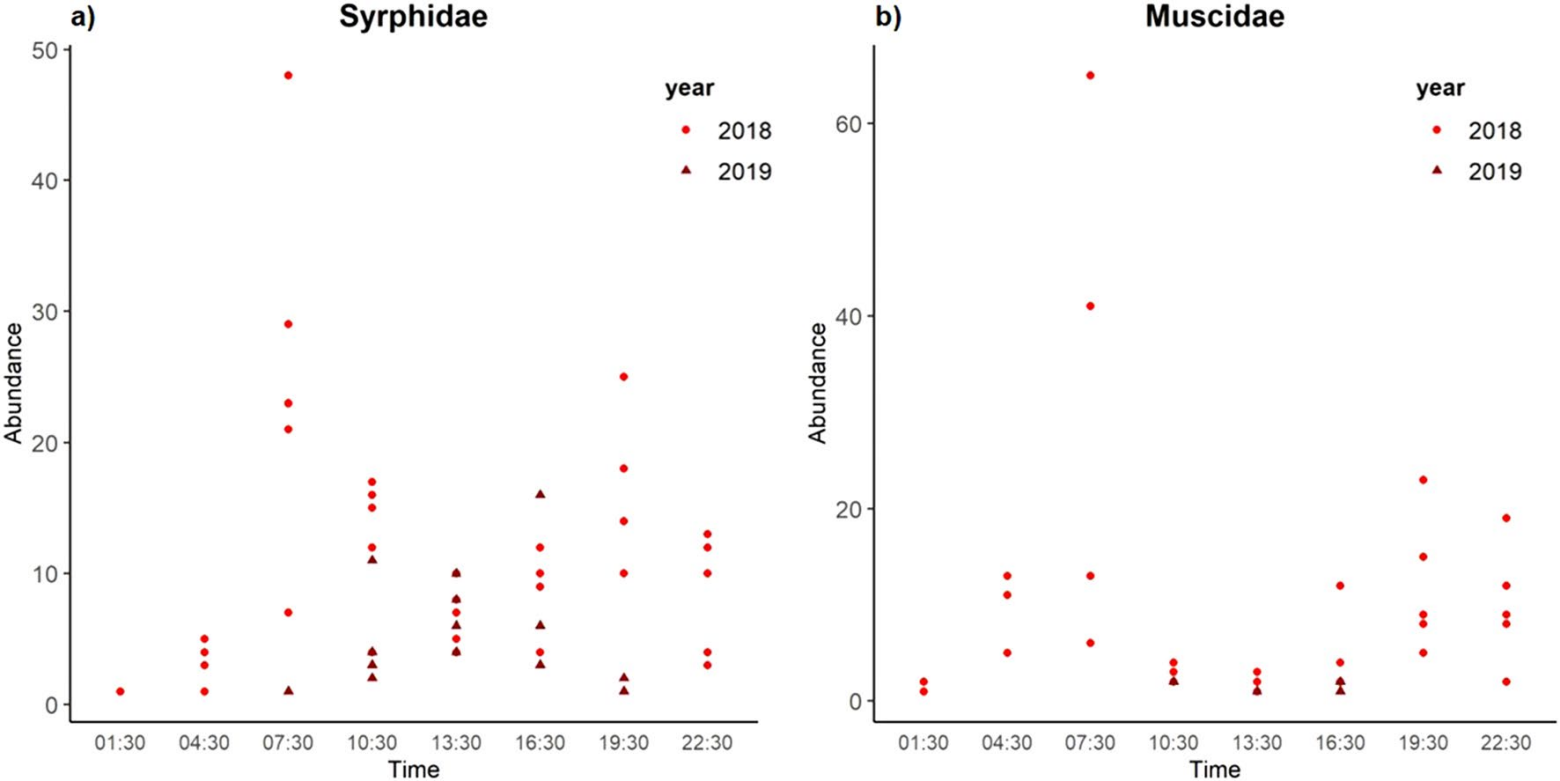
Abundance across the 24-h cycle of the two most abundant Diptera families (**a**) Syrphidae and (**b**) Muscidae in the years 2018 and 2019.

Both Diptera and Hymenoptera exhibited a robust pattern of diel foraging activity, with peak activity during the day. We observed few individuals of Lepidoptera (Table 2) and almost all were observed during the day. The activity patterns of Diptera and Hymenoptera differed significantly from each other in 2018 (W = 11.733, p < 0.001) but not in 2019 (W = 0.3019, p = 0.5366). In 2018, the diel activity pattern of Diptera and Hymenoptera followed a bimodal distribution, with the largest peak at 07:30 and a smaller peak at 19:30. Between 07:30 and 19:30 there was a sharp drop in foraging activity, which was steeper in Diptera than Hymenoptera. Activity of both Diptera and Hymenoptera in 2018 was lowest at 01:30, but never dropped to zero (Fig. 2). Wald tests comparing activity at subsequent sampling times are provided in Supplementary Table S2. The abundance of Syrphidae and Muscidae followed the same pattern as the activity on the order level (Fig. 3).

In 2019, the diel foraging activity of both Diptera and Hymenoptera followed a unimodal distribution, with peak activity in both orders between 10:30 and 16:30. Between 22:30 and 07:30 the foraging activity dropped to near zero or zero (Fig. 2). At the family level, Syrphidae abundance showed a unimodal pattern, with peak abundance around solar noon and no observations between 22:30 and 04:30. Muscidae were recorded only between 10:30 and 16:30 (Fig. 3). The overlap in foraging activity patterns was greater across different taxa in the same sampling year (overlap-index between Diptera and Hymenoptera in 2018: dhat4 = 0.803 and in 2019: daht4 = 0.915) than within the same taxa across the sampling years (Diptera: dhat4 = 0.429; Hymenoptera: dhat4 = 0.706). The abundance of pollinators was significantly explained by hour of sampling and temperature (Table 3). Abundance of pollinators showed the expected hump-shaped relationship with temperature, although we note that all high temperature data were observed in 2018 (Table 3, Supplementary Fig. S4).

**Table 3:** The effect of hour of sampling and temperature on the abundance of pollinators.

	Estimate	SE	z-value	p-value
Intercept	0.753	0.087	8.702	**< 0.001**
Sine (sampling hour)	0.689	0.033	20.651	**< 0.001**
Cosine (sampling hour)	0.006	0.028	0.214	0.83
Temperature^2^	0.117	0.004	29.985	**< 0.001**

Significant effects are printed in bold.

## Discussion

We found evidence for robust patterns in diel foraging activity for the most common pollinator orders, and across the two most abundant families of Diptera, Syrphidae and Muscidae. Peak foraging activity occurred during the day, with less to no activity during the night. The abiotic conditions at night during our sampling would allow for foraging (*Bombus terrestris* workers for example have been observed foraging at temperatures as low as 3 °C^20^). There is also no obvious change in floral resource availability from day to night (i.e., flowers on our transect did not close at night, however, we note that we did not measure the availability of floral rewards, which could vary between day and night). The most likely explanation is, that pollinators focus their foraging activity on the times when temperatures are close to their thermal optima.

Our observations contrast with the findings of a study conducted at a 24-h grain in the Swiss Alps, where insect visitation rate never dropped to zero and moths were abundant nocturnal flower visitors^4^. During the Arctic summer, moths retain a distinct diel periodicity and are active at night^14^. Historical observations made in our study region in July 1896–1897 also show the presence of multiple species of noctuid moths (family Noctuidae) interacting with the moth-pollinated plant *Silene vulgaris*^21^. Despite projections suggesting the abundance of moths and butterflies will increase at high latitudes under climate change^22^ and the high abundance of *S. vulgaris* at our site, we observed only few Lepidoptera and no Noctuidae. At this point, we are not able to present a satisfying explanation for this.

We found a marked difference in diel foraging activity patterns across years. In the average temperature year, activity was highest during noon. This unimodal pattern found in the average temperature year is in line with other observations on diel flower visitor activity in the Scandinavian mountains^9^. In the high temperature year on the other hand, pollinator activity drastically dropped around noon. This decline in activity in the hottest part of the day in 2018 is likely explained by the extreme temperatures, as temperature significantly explained pollinator abundance in our model. Anthophilous insects often cease their foraging activity during the warmest times of day to seek out cooler microhabitats and avoid overheating^23^ and these behavioral changes are known to have fitness costs^23^. In contrast to 2019, the foraging activity of both Diptera and Hymenoptera in 2018 never ceased completely at night. Hence, it is possible that warmer temperatures and more frequent temperature extremes due to climate change could potentially lead to novel diel activity patterns. As extreme temperatures inhibit pollinator activity around noon, diurnal pollinators might be forced to shift activity to night time to compensate for the lost foraging window.

We found significant differences in abundance and composition of most pollinator families across the sampling years. Flower visitors were 67% less abundant in 2019, the year following record heat year 2018. Diptera displayed the lowest interannual overlap in diel activity and flies, especially muscid flies, also underwent a large interannual decrease in abundance. Insect populations are highly dynamic^24, 25^ and our results could reflect normal interannual variation. Alternatively, our results could suggest that pollinators, particularly flies, in our system are climate sensitive. The higher abundance of flies in 2018 could reflect benefits of the heat, allowing improved growth, activity and reproduction of organisms that are usually cold-limited^8^. Conversely, the low abundance in 2019 might results from population declines in response to the physiological and behavioral stress from the 2018 heatwave on flies. Several insect species, such as flour beetles and honey bees, have been shown to suffer from heavily reduced male reproductive success when exposed to heatwave conditions^26, 27^. Organisms at high latitudes or altitudes may be particularly vulnerable when temperatures exceed their thermal optima during a heatwave. For example, a bumblebee species occurring at low altitudes can tolerate air temperatures of up to 5° warmer than those occurring at high altitudes^28^. Further, our results could reflect differences in phenology and resource availability across the years. Changes in the identity of the seven most visited plant species across the years indicate that it is possible that the plant community might have been in an advanced phenological stage in 2018 compared to 2019. Plant resources are known to change in composition and concentration in response to high temperatures^29, 30^.

In many Alpine and Arctic areas, muscid flies have been identified as the most common flower visitors^31^ and key pollinators for certain plant species^32^. Despite this, Muscidae remain largely understudied. Globally, there are over 5000 accepted species of Muscidae^33^ and over 300 of these are present in Finland^34^. However, none of these species have been assessed by the IUCN red list for extinction threat^34^. Recently, concerns have been raised about the declining numbers of muscid flies in Arctic areas and the potential to impair ecosystem services^17, 25, 35^. More research is needed investigating the thermal sensitivity and the longer-term temporal fluctuations of muscid flies.

Most pollination studies do not assess nocturnal pollinators and thus do not look for trends that might inform on patterns of their decline. Our temporal sampling is designed for sampling nocturnal pollinators, and their absence is concerning given known historical records^21^. The absence of moths might have potential implications for the pollination of plant species and highlights the need for future work in pollination ecology to incorporate pollinators across all 24 h. To establish if this is a general problem that should be of conservation concern, other locations with baseline data should be sampled for diel patterns in nocturnal pollinators.

To our knowledge, our study is the first to address patterns of diel foraging activity of pollinators on a community level in the Arctic summer and our results contribute to the growing knowledge of pollination in the Arctic. There is a need for long term observations of pollinators, plants and their interactions at high latitude sites, especially because climate change in these regions is progressing particularly rapidly^36^. Furthermore, there is a need for experimental data to assess consequences of changing plant–pollinator interactions for plant reproduction (i.e. pollen limitation), as our understanding of the extent and magnitude of pollen limitation at high latitudes is currently limited^37^ (but see^38, 39^).

## Conclusion

Here, we show evidence of strong temporal variation at two temporal grains in an Arctic ecosystem. Despite the constant daylight and warm temperatures in the Arctic summer, we find robust diel foraging activity patterns for several taxa of pollinators. Further, there were significant differences between years in the abundance, composition and diel activity patterns of pollinators, likely in response to extreme heat in 2018. Diptera and especially muscid flies, which are important pollinators in the Arctic, showed stark differences in activity between years and much lower abundance in the year following the extreme heat event. This potential sensitivity of muscid flies and the absence of nocturnal Lepidoptera observed here raise conservation concerns not only for these groups, but also for the plant species that rely on them for reproduction.

## Methods

### Sampling location and dates

Data collection took place between 10 and 20 July in 2018 and 09 and 18 July in 2019, in the proximity of the town of Kittilä, Finland (67.655465°N, 24.912411°E, 178 m), located ~ 120 km north of the Arctic Circle. From May 29 until July 16 the sun does not set in Kittilä, and civil twilight only occurs before May 6 and after August 8. The landscape around Kittilä is dominated by boreal forest and has low human population density, few invasive plant species and little agricultural land use. A transect was established on a ruderal meadow selected to contain both typically day and night pollinated plant species. In both sampling years, we performed a vegetation survey along the transect. Abundant plant species included *Tanacetum vulgare* (Asteraceae), *Heracleum sphondylium* (Apiaceae), *Trifolium pratense* (Fabaceae) (diurnally pollinated) and *Silene vulgaris* (Caryophyllaceae) (nocturnally pollinated^40^). Flowering plant species richness was similar in the two sampling years (species totals n = 19 in 2018 and n = 18 in 2019). For the seven most visited plant species of each sampling year, we obtained data on flowering season using the database BiolFlor^41^.

### Data collection

During 15 min observation periods (excluding handling time), all active flower visitors along a 30 × 2 m transect were observed. An active flower visitor was defined as any individual belonging to the orders Diptera, Hymenoptera or Lepidoptera that intentionally moved on a flower thereby touching the reproductive organs of the flower. Here, we refer to flower visitors and pollinators synonymously, although we realize that not all flower visitors are efficient pollinators. If possible, pollinator species were identified in the field. When direct identification was not possible, the specimens were collected by net for later identification in the lab. All individuals were identified to at least family level. The transect was sampled every 3 h over a 24-h cycle, starting at 01:30 (EEST) (astronomical midnight, sun at lowest point), resulting in eight sampling rounds per 24-h cycle. Sampling took place on days with favourable weather (no rain, low wind), if possible on consecutive days, to minimize the effects of seasonal turnover. The data collection was repeated for 5 full 24-h cycles in each year. Abiotic factors with potential impact on pollinator activity, namely global solar radiation (the total short-wave radiation from the sky falling onto a horizontal surface on the ground, including direct solar radiation and diffuse radiation), air temperature and wind speed were obtained in an hourly interval for the entire sampling period from the nearest weather station. Specifically, wind speed and air temperature were obtained from Kittilä kirkonkylä (67.65210°N, 24.90162°E; 181 masl) and global solar radiation from Sodankylä Tähtelä (67.36663°N, 26.62901°E; 179 masl)^42^. Kittilä kirkonkylä is located around 600 m from our sampling site, while Sodankylä Tähtelä is located 80 km from our sampling site. Sodankylä Tähtelä is at a similar latitude and thus the daily rhythmicity of solar radiation is comparable to our sampling site. However, we note that this might not be close enough to capture temporal variation in solar radiation due to could cover.

### Statistical analysis

All statistical analyses were conducted in R version 3.6.0^43^.

### Abiotic conditions across years

We performed t-tests (using *t.test* from the R-package *stats*^43^) to compare the mean values of abiotic factors (temperature, global solar radiation and wind speed) recorded during our sampling times between years (sample size of each abiotic factor in each year: n = 40: 5 days × 8 sampling rounds).

### Abundance and community composition

We compared the mean abundances of our focal pollinator taxa, as well as the number of floral units along the transect between sampling years using t-tests (*t.test* from package *stats*). To estimate ecological dissimilarity of the pollinator community of each sampling day we calculated the Jaccard similarity index^44^ and the Bray–Curtis dissimilarity index^45^ using the package *vegan*^46^. To visualize pollinator assemblages between years, we used a non-metric multi-dimensional scaling (NMDS) ordination based on Jaccard similarity index and Bray–Curtis dissimilarity index using *metaMDS* from the *vegan* package. We performed a permutational multivariate analysis of variance (PERMANOVA) (using *adonis* from package *vegan*) to statistically test if the community composition differed between the sampling years.

### Pollinator activity

We compared the activity of pollinators across the 24-h cycle using the package *activity*^47^. To fit activity models to our observation data for the most abundant pollinator orders (Diptera and Hymenoptera) we used the function *fitact.* The *fitact* function fits a kernel density to observational data from radian time of day and estimates the activity level from the resulting circular kernel distribution, which is a nonparametric representation of the probability density function of a random variable. Since our observations were not made continuously, but rather in 3 h-intervals, we adjusted the kernel bandwidth in each model to smoothen the circular kernel distribution. The bandwidth value was chosen by visually checking the best fit of the circular kernel distribution to our data. Confidence limits were generated by bootstrapping the fitted distribution 1000 iterations. The fitted circular kernel distributions and confidence intervals were plotted using *plot.actmod*. To estimate the overlap of the diel activity patterns, we calculated the dhat4 overlap index between the fitted circular kernel distributions (see^48^) using *ovl4*. Overlap was estimated within each taxon between the sampling years, as well as across taxa within the same sampling year. We tested for the statistical difference between the activity level estimates of Diptera and Hymenoptera using *compareAct*. To test for the statistical difference in activity levels at our sampling times we used *compareTimes*. *CompareAct* and *compareTimes* perform Wald tests (see^49^) to test for the statistical difference between two or more activity level estimates. Lepidoptera were excluded from these analyses due to low sample size. For the two most abundant Diptera families (Muscidae and Syrphidae), we present raw data on abundance across the 24-h cycle, since the low sample size did not allow us to reliably fit activity models to the data.

### Effects of abiotic factors on pollinator abundance

Due to concerns of collinearity of the abiotic factors and time of day, we first determined the correlation coefficients between the different abiotic factors and time of day. To account for cyclical nature of time of day, we transformed hour of sampling to radian and performed circular-linear correlations (*circlin.cor* from package *Directional*^50^. Collinearity of predictor variables can inflate the variance of regression parameters and potentially lead to a wrong identification of relevant predictors in a statistical model. If correlation coefficients between predictor variables are > 0.7, collinearity begins to severely distort model estimation^51^. In our case, the correlation coefficient between time of day and global solar radiation exceeded 0.7 (Supplementary Table S3), thus we excluded global solar radiation as a predictor variable. We also did not include wind speed as predictor variable, since our observations of wind speed were all in a narrow range between one and two meters per second. We proceeded to perform a regression analysis, specifically, we ran a generalised linear model assuming a Poisson distribution (using *glm* from package *stats)*, including pollinator abundance as response variable and temperature and hour of sampling as explanatory variables. In order to account for the circular nature of time of day, the variable hour of sampling was transformed to radian and included in the model as function of the sine and cosine. Due to the expected hump-shaped relationship between temperature and pollinator abundance, temperature was fitted as a quadratic function. We note that all temperature values between the vertex and maximum of the parabola are from the year 2018 (Supplementary Fig. S4).

## Supplementary information

Supplementary Information.

## Data Availability

The datasets generated during and/or analysed during the current study are available from the corresponding author on reasonable request.
