# Supplementary material for: Diel-scale temporal dynamics in the abundance and composition of pollinators in the Arctic summer
Source: Sci Rep. 2020 Dec 3;10:21187. doi: 10.1038/s41598-020-78165-w (PMC7713049; doi:10.1038/s41598-020-78165-w)
Supplement: Supplementary file 1 — Supplementary Information. [file 41598_2020_78165_MOESM1_ESM.pdf]

Diel-scale temporal dynamics in the abundance and composition of pollinators in the Arctic summer

Leana Zoller[1,2*]

Joanne M. Bennett[1,2,3]

Tiffany M. Knight[1,4,2]

1. Institute of Biology, Martin Luther University Halle-Wittenberg, Am Kirchtor 1, 06108 Halle (Saale), Germany

2. German Centre for Integrative Biodiversity Research (iDiv) Halle-Jena-Leipzig, Deutscher Platz 5e, 04103 Leipzig, Germany

3. Centre for Applied Water Science, Institute for Applied Ecology, Faculty of Science and Technology, University of Canberra, Bruce, ACT 2617, Australia

4. Department of Community Ecology, Helmholtz Centre for Environmental Research- UFZ, Theodor-Lieser-Straße 4, 06120 Halle (Saale), Germany

Supplementary figures and tables

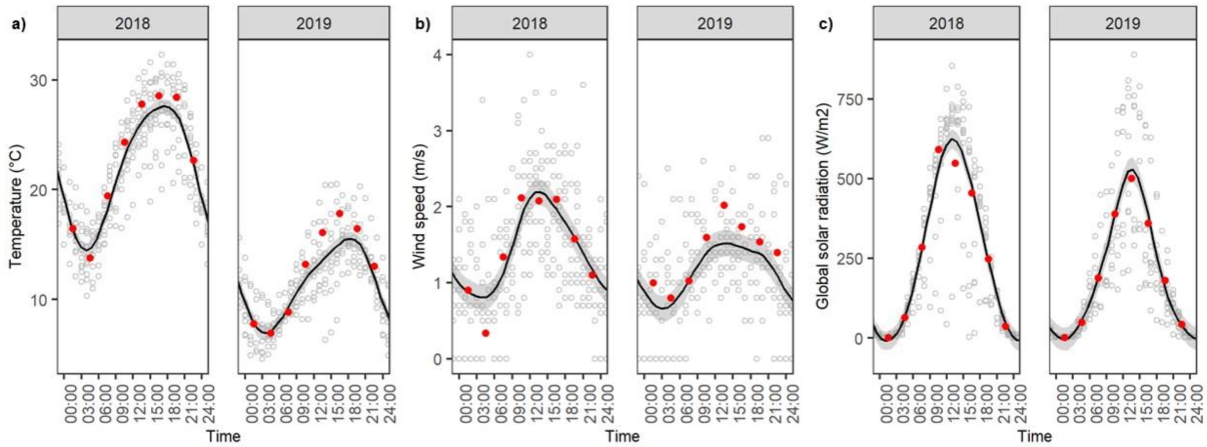

Supplementary Figure S1. *Abiotic factors* ***a)*** *temperature,* ***b)*** *wind speed and* ***c)*** *global solar radiation over the diel cycle in the years 2018 and 2019. Red points depict the mean of values measured at the beginning of each sampling round. Grey circles indicate hourly measurements during our entire sampling period. The black line depicts the loess regression fitted through the hourly data points. Raw data were obtained from the Finnish Meteorological institute (FMI). Measures of temperature and wind speed stem from the weather station Kittilä kirkonkylä and measures of global solar radiation were taken at the weather station Sodankylä Tähtelä.*

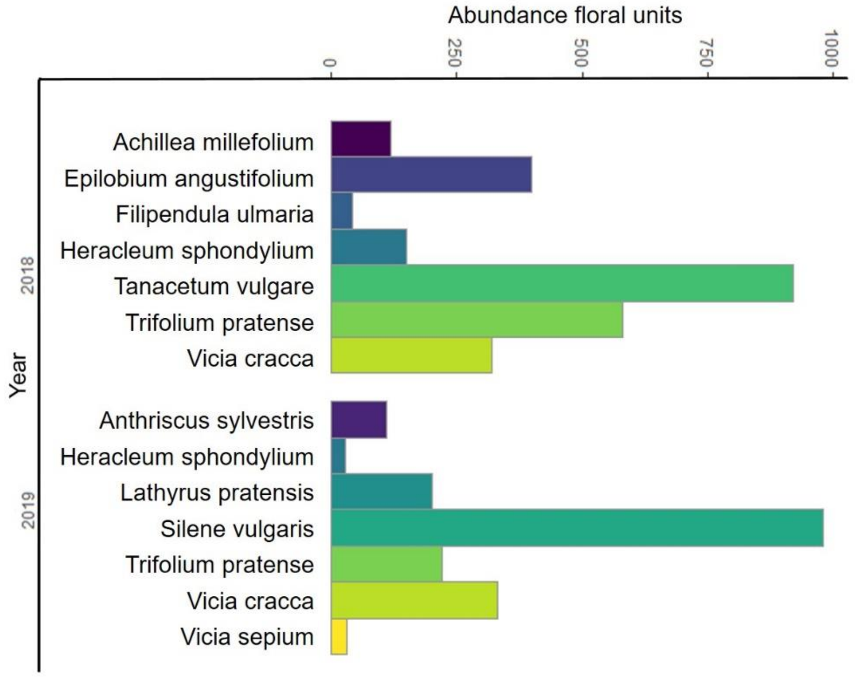

Supplementary Figure S2. *Identity and abundance of floral units (number of flowers or inflorescences pre 30x2 m transect) of the seven most visited plant species in the years 2018 and 2019.*

Supplementary Table S1. *The most visited plant species in 2018 and/or 2019 and information about their flowering season. Plant species with the earliest start of flowering (May) were only on the list of most visited species in the year 2019, whereas those with the latest start of flowering (July) were only on the list of most visited species in the year 2018 (source: Database BiolFlor).*

Plant species	flowering season	year most visited
Achillea millefolium	June – Oct.	2018
Anthriscus sylvestris	May – Aug.	2019
Epilobium angustifolium	July – Aug.	2018
Filipendula ulmaria	June – Aug.	2018
Heracleum sphondylium	June – Sept.	2018, 2019
Lathyrus paratensis	June – Aug.	2019
Silene vulagris	May- Sept.	2019
Tanacetum vulgare	July – Sept.	2018
Trifolium pratense	June – Sept.	2018, 2019
Vicia cracca	June – Aug.	2018, 2019
Vicia sepium	May - June	2019

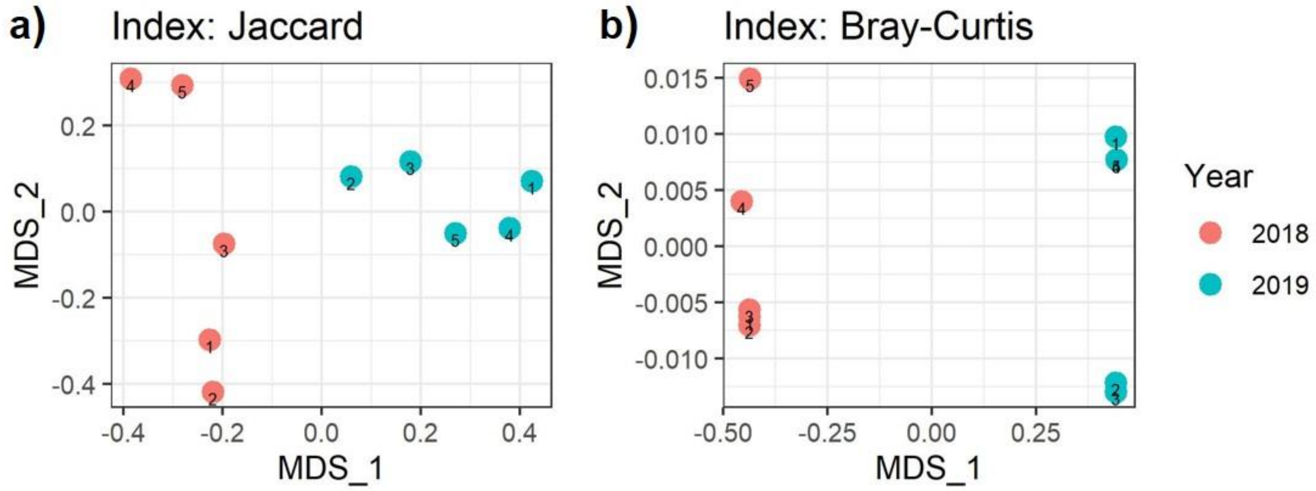

Supplementary Figure S3*. Visualization of non-metric multidimensional scaling (NMDS) ordination based on* ***a)*** *Jaccard similarity index and* ***b)*** *Bray-Curtis dissimilarity index of the pollinator assemblages in the years 2018 and 2019. Each point represents one of the five 24-hour sampling cycles.*

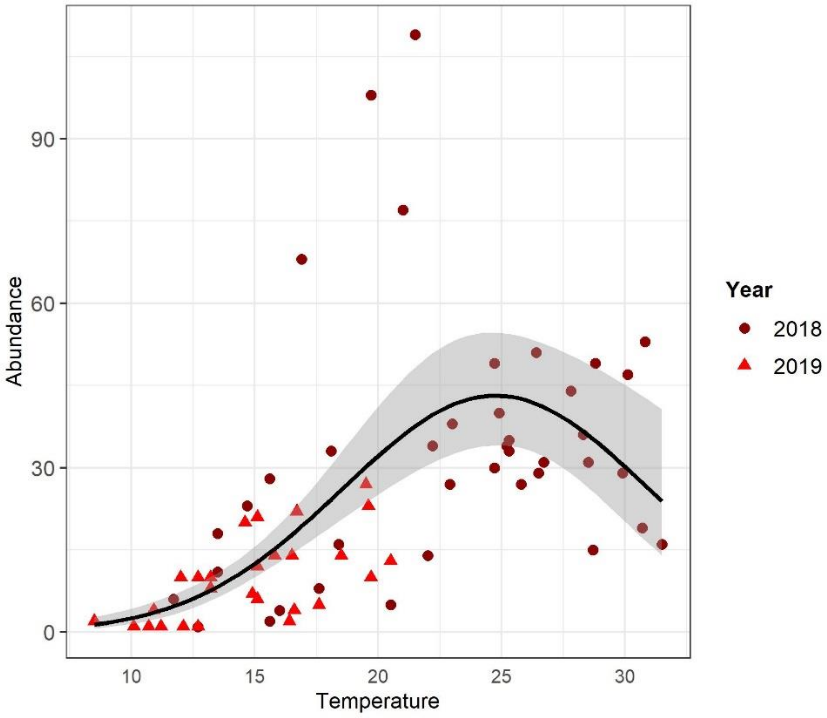

Supplementary Figure S4*. Relationship between abundance of pollinators and temperature. The quadratic function is fit using all data. The years are color-coded in the figure to illustrate that high temperature observations come only from year 2018.*

Supplementary Table S2. *Results of wald-tests pairwise comparing activity models at subsequent sampling times (01:30, 04:30, 07:30, 10:30, 13:30, 16:30, 19:30 and 22:30) for the orders Diptera and Hymenoptera in the two sampling years 2018 and 2019. Significant values are printed in bold.*

Diptera 2018				
Time	Difference	SE	W	p
01:30-04:30	-0.0910	0.0172	27.972	**<0.001**
04:30-07:30	-0.2627	0.0271	94.001	**<0.001**
07:30-10:30	0.2366	0.0279	71.900	**<0.001**
10:30-13.30	0.0718	0.0198	13.166	**<0.001**
13:30-16:30	-0.0396	0.0180	4.812	**0.028**
16:30-19:30	-0.1152	0.0228	25.542	**<0.001**
19:30-22:30	0.0752	0.0237	10.041	**0.0015**
Diptera 2019				
01:30-04:30	0.0062	0.0098	0.397	0.529
04:30-07:30	-0.0469	0.0191	6.024	**0.014**
07:30-10:30	-0.2269	0.0374	36.913	**<0.001**
10:30-13.30	-0.1562	0.0501	9.712	**0.002**
13:30-16:30	0.092	0.052	3.128	0.077
16:30-19:30	0.2074	0.0437	22.515	**<0.001**
19:30-22:30	0.0988	0.0299	10.894	**<0.001**

Hymenoptera 2018				
Time	Difference	SE	W	p
01:30-04:30	-0.0522	0.0153	11,602	**<0,001**
04:30-07:30	-0.1788	0.0249	51,411	**<0,001**
07:30-10:30	0.0533	0.0274	3,777	0,052
10:30-13.30	0.0283	0.0244	1,350	0,245
13:30-16:30	-0.0261	0.0244	1,141	0,285
16:30-19:30	-0.0029	0.0246	0,004	0,907
19:30-22:30	0.1150	0.0220	27,356	**<0,001**
Hymenoptera 2019				
01:30-04:30	0.0044	0.0099	0.193	0.66
04:30-07:30	-0.0353	0.0196	3.238	0.072
07:30-10:30	-0.1827	0.0404	20.483	**<0.001**
10:30-13.30	-0.1765	0.0586	9.077	**0.003**
13:30-16:30	0.0549	0.0613	0.802	0.37
16:30-19:30	0.1202	0.0537	5.011	**0.025**
19:30-22:30	0.1572	0.0422	13.844	**<0.001**

Supplementary Table S3. *R^2 and R values obtained by running correlations between radian time of day and abiotic factors air temperature, global solar radiation and wind speed.*

Variables	R^2	R
radian time –air temperature	0.241	0.491
radian time – solar radiation	0.580	0.761
radian time – wind speed	0.211	0.459
